# Supplementary material for: Conserved Sequences in the 5′ and 3′ Untranslated Regions of Jingmenvirus Group Representatives
Source: Viruses. 2023 Apr 15;15(4):971. doi: 10.3390/v15040971 (PMC10141212; doi:10.3390/v15040971)
Supplement: Supplementary file 1 [file viruses-15-00971-s001.zip › viruses-2299143-supplementary.pdf]

Table S1. Oligonucleotides used for virus amount estimation of ALSV and YGTV

Virus	Primer	Sequence
ALSV	Miass_gly_3F	5` TGGATCAGCTCACACCACAC 3`
	Miass_gly_3R	5` TCACCGTCACAGTGGAATGG 3`
YGTV	Yanggou_gly_1F	5`ACTACTGGTTGCCGTCCTCG 3`
	Yanggou_gly_1R	5`ACTACTGGTTGCCGTCCTCG 3`

Table S2. Oligonucleotides used for RACE PCR

Primer	Sequence	Direction	Virus	segment
Miass_NS5_RACE	5` GCCAACACTATCAGGACAATCATG 3`	Reverse	Alongshan	1
Alonshan_seg2_492r	5` TCCGTTTGGTTGTGCCGAT 3`	Reverse	Alongshan	2
Miass_NS3_RACE	5` CAATGCCATGATCGCTAGTCC 3`	Reverse	Alongshan	3
Miass_C_RACE	5` GTCCCAGCAGCTCGATCT 3`	Reverse	Alongshan	4
Miass_NS5_3F	5` AGGCCATGAATGCAACAGGA 3`	Forward	Alongshan	1
Miass_gly_1F	5` CCACATCACGGGAGGTATCG 3`	Forward	Alongshan	2
Miass_NS3_1F	5` AGGAGAGGGCCATCAGGAAT 3`	Forward	Alongshan	3
Miass_C_2F	5` CACGGGAGAAACGGATAGGG 3`	Forward	Alongshan	4
Yanggou_seg1_612r	5` TCTGCCATCCATTCTTCCT 3`	Reverse	Yanggou	1
Yanggou_gly_1R	5` GTCGCTGCAGTCAAATATCT 3`	Reverse	Yanggou	2
Yanggou_seg3_772r	5` CGTACCTTCTGTCCTCTATCCA 3`	Reverse	Yanggou	3
Yanggou_seg4_1R	5` CATTCTTCGGATGTCGGCCT 3`	Reverse	Yanggou	4
Yanggou_seg1_3F	5` ATTGCAGGATGGGACACCAA 3`	Forward	Yanggou	1
Yanggou_seg2_4F	5` GGATGGGGTGGTCAGAGTC 3`	Forward	Yanggou	2
Yanggou_seg3_3F	5` GCGTTGTCACCACCTCGATA 3`	Forward	Yanggou	3
Yanggou_seg4_3F	5` CTGGTGGCATCACTGGATGT 3`	Forward	Yanggou	4

Table S3. Positive clone ratios for Alongshan and Yanggou viruses

Virus	Segment	Bacterial clones taken	
		with intact 3`UTR	total
YGTV strain Bredy15-22181	3	4	7
YGTV strain Bredy15-22188	2	2	4
	3	2	2
YGTV strain Bredy15-22189	2	2	3
	3	7	10
YGTV Plast15-22438	3	2	5
ALSV Miass502	1	0	16

Table S4. Oligonucleotides used for Sanger sequencing PCR products obtained after RACE.

Primer	Sequence	Direction	Virus	segment
Miass_NS5_RACE	5` GCCAACACTATCAGGACAATCATG 3`	Reverse	Alongshan	1
Alonshan_seg2_492r	5` TCCGTTTGGTTGTGCCGAT 3`	Reverse	Alongshan	2
Miass_NS3_RACE	5` CAATGCCATGATCGCTAGTCC 3`	Reverse	Alongshan	3
Miass_C_RACE	5` GTCCCAGCAGCTCGATCT 3`	Reverse	Alongshan	4
Miass_NS5_3`RACE	5` AAAGCCAGCATGTCACTTGG 3`	Forward	Alongshan	1
Miass_Gly_3`RACE	5` TCTTCCGAGCTAACATCCCC 3`	Forward	Alongshan	2
Miass_NS3_1F	5` AGGAGAGGGCCATCAGGAAT 3`	Forward	Alongshan	3
Miass_C_3`RACE	5` CCAGTGTCTTGGCATTCTCG 3`	Forward	Alongshan	4
Yanggou_seg1_469r	5` TGAGATCCATCAGCACTCTG 3`	Reverse	Yanggou	1
Yanggou_seg2_350r	5` GAGGACGGCAACCAGTAGT 3`	Reverse	Yanggou	2
Yanggou_seg3_535r	5` AGCTGATGGGCTGTAGATAAC 3`	Reverse	Yanggou	3
Yanggou_seg4_395r	5` CCACGGCTCTTGTCCAC 3`	Reverse	Yanggou	4
Yanggou_seg1_2495f	5` TATAGGGATGACTCAACTCAGGA 3`	Forward	Yanggou	1
Yanggou_seg2_2182F	5` CATTGAAGAACTACTATTTGTAC 3`	Forward	Yanggou	2
Yanggou_seg3_2348F	5` AGAGGACGAGGCAGAACAG 3`	Forward	Yanggou	3
Yanggou_seg4_2293F	5` CTCAGCATGTATAGTGATGGT 3`	Forward	Yanggou	4

Table S5. Oligonucleotides used for Sanger sequencing PCR products after cloning in pCR2.1 vector

Primer	Sequence	Designed by
M13 (-21) Primer	5′-TGTAAAACGACGGCCAGT-3′	Applied Biosystems
M13_long_F	5′-ACAGGAAACAGCTATGACCATG-3′	custom

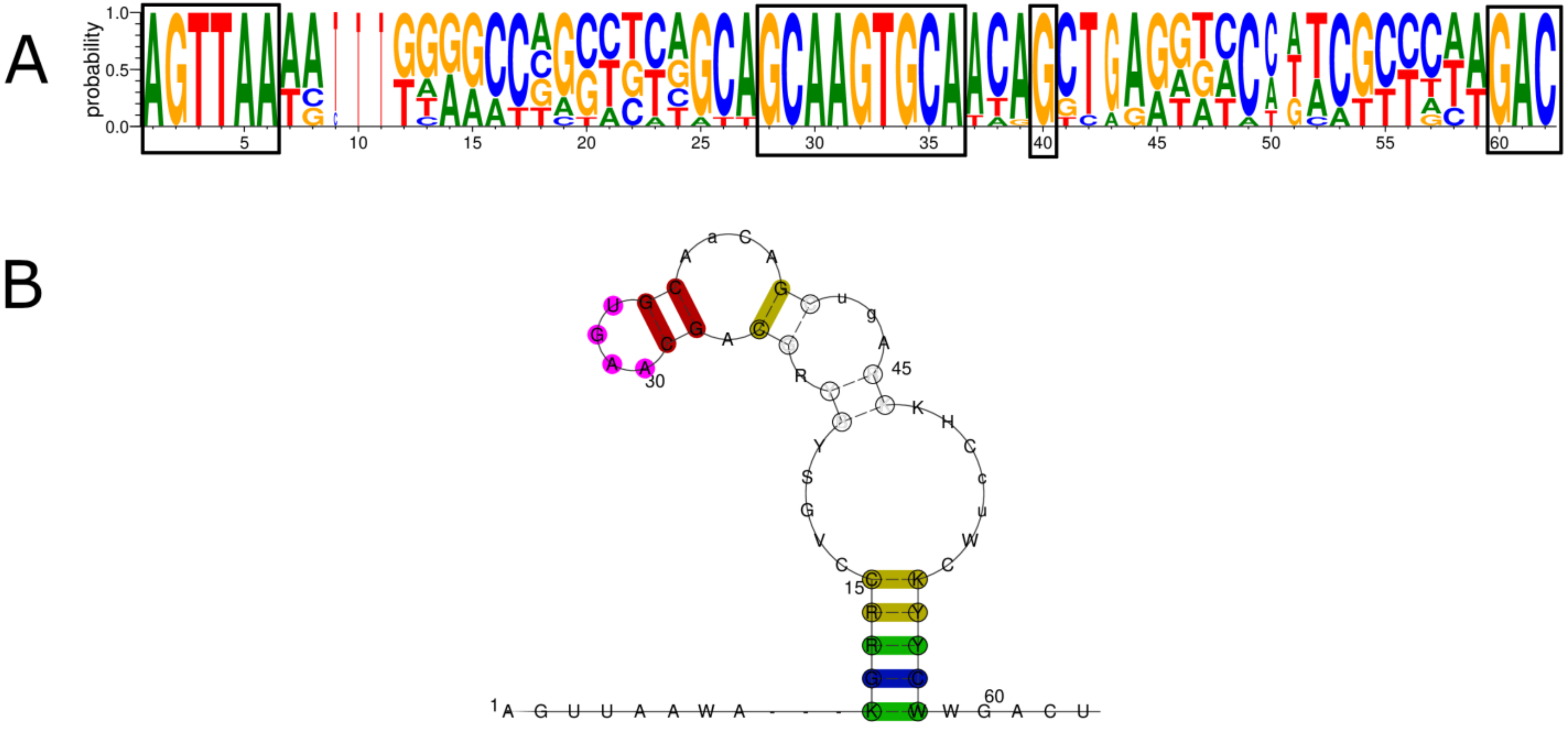

Figure S1. Consensus sequences of the ALSV and YGTV 5′UTRs (A) according to ClustalW alignment and structures predicted with RNAalifold (B) based on the consensus sequences

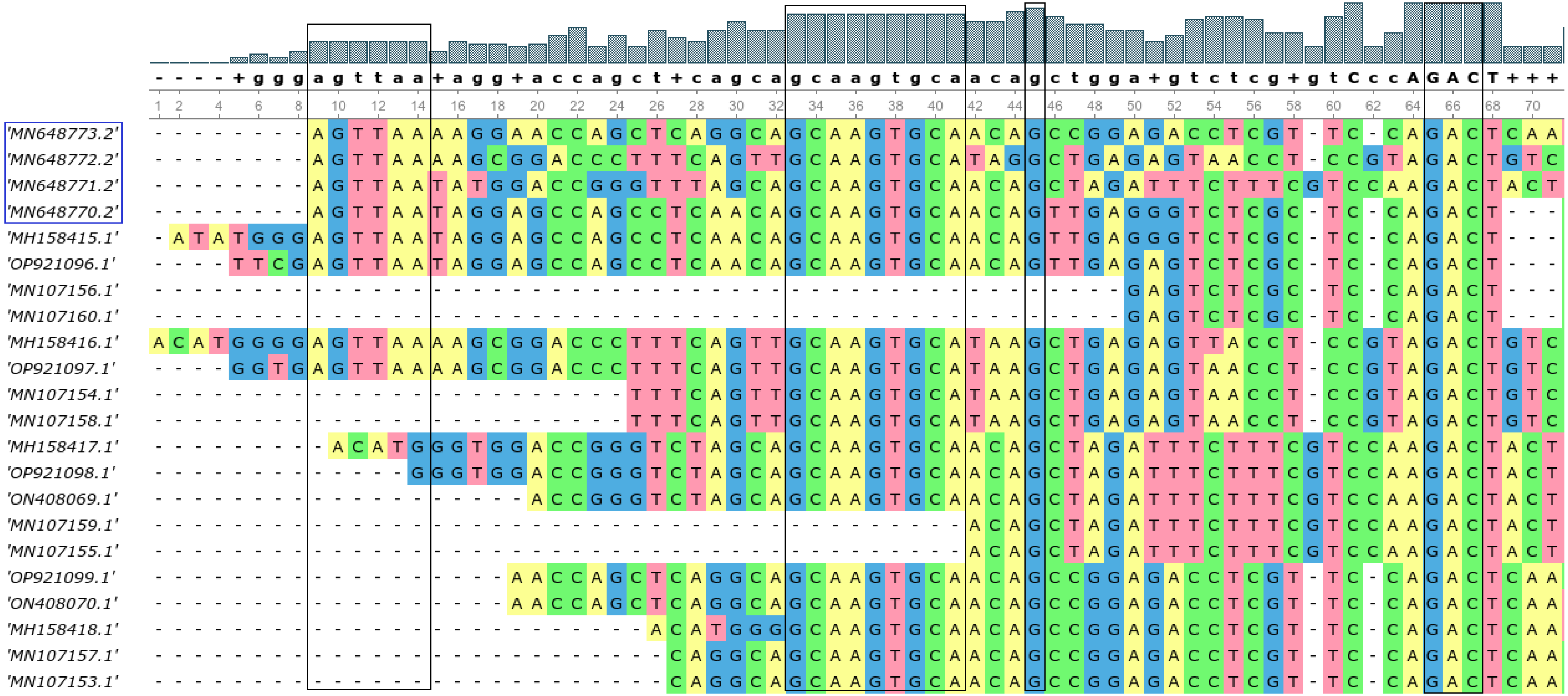

Figure S2. Conserved sequences in the 5′UTRs of ALSV genomes found in GenBank according to ClustalW alignment. Regions discussed in the article are in black boxes. Sequences used as references during alignment construction are marked by blue boxes

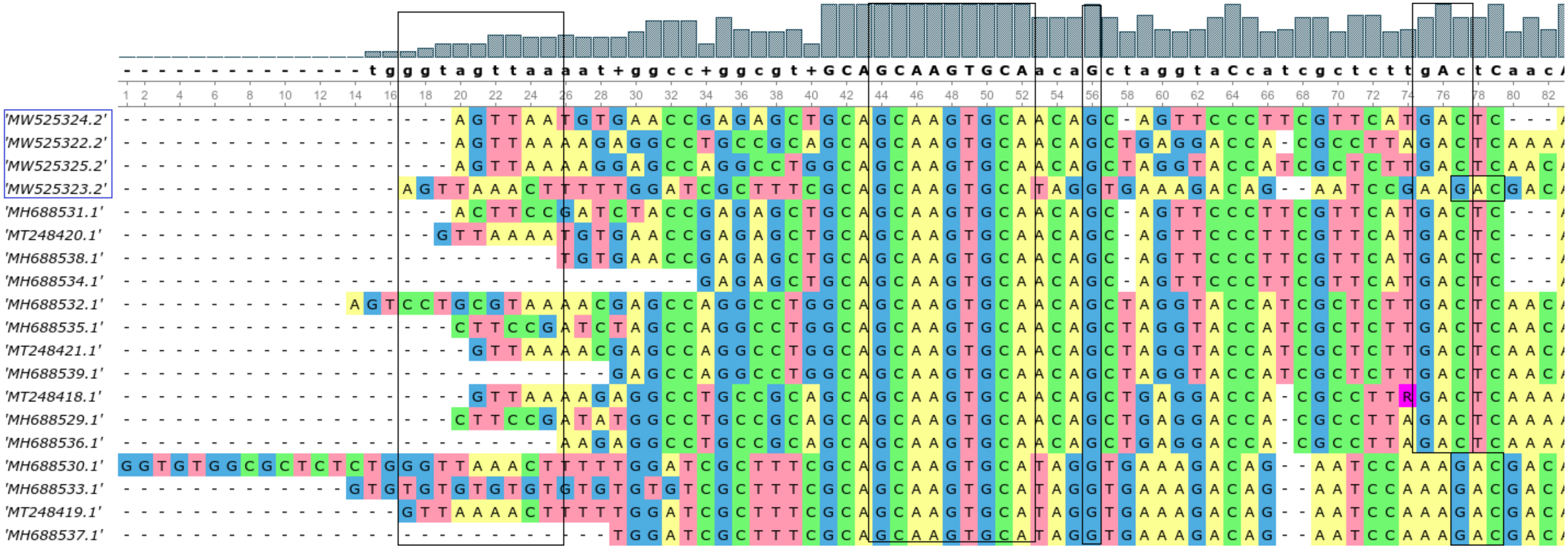

Figure S3. Conserved sequences in the 5′UTRs of YGTV genomes found in GenBank according to ClustalW alignment. Regions discussed in the article are in black boxes. Sequences used as references during alignment construction are marked by blue boxes

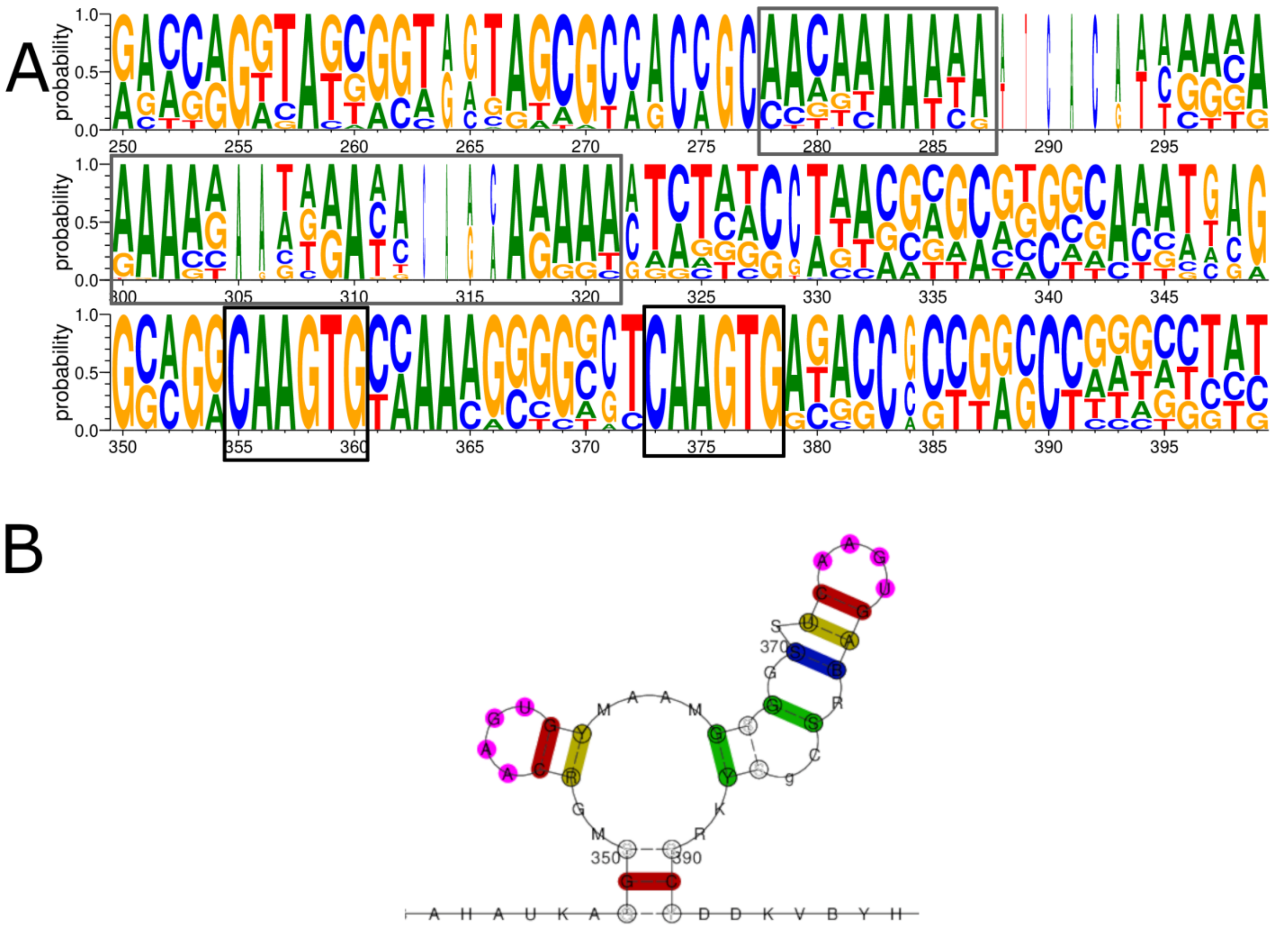

Figure S4. Consensus sequences of ALSV and YGTV 3′UTRs (A) according to ClustalW alignment and structures predicted by RNAalifold (B) based on the consensus sequences

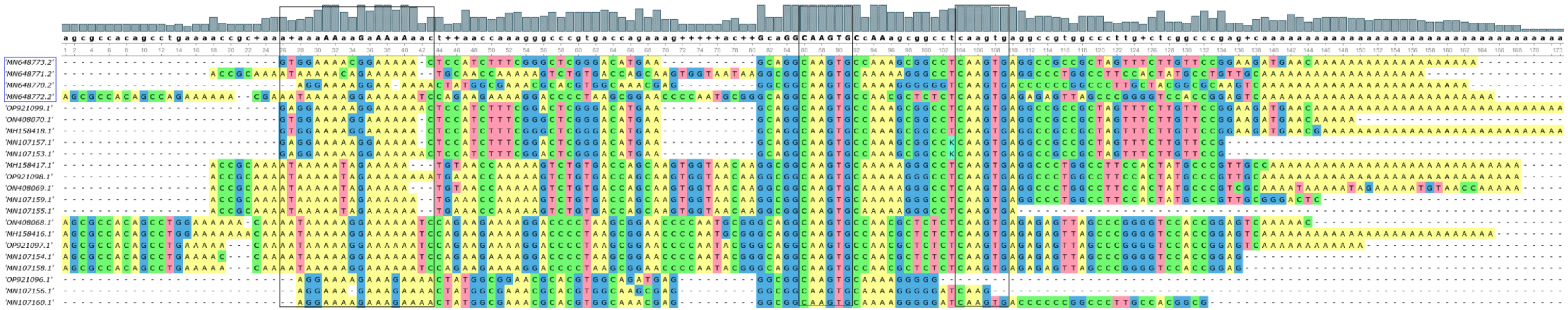

Figure S5. Conserved sequences in the 3′UTRs of ALSV genomes found in GenBank according to ClustalW alignment

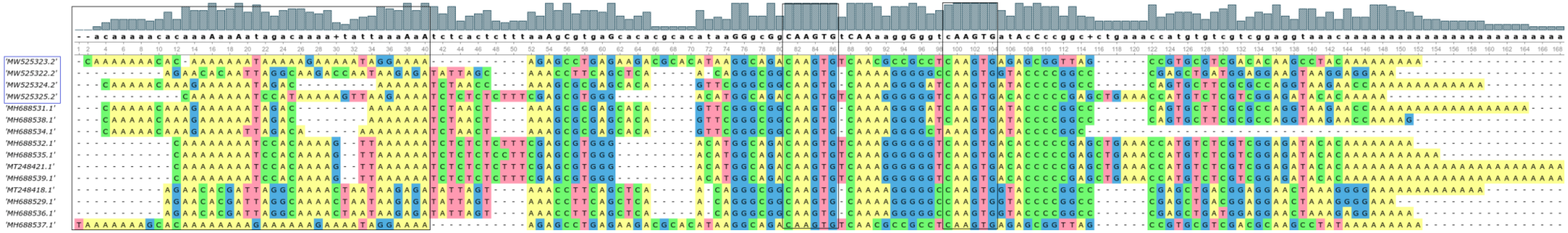

Figure S6. Conserved sequences in the 3′UTRs of YGTV genomes found in GenBank according to ClustalW alignment

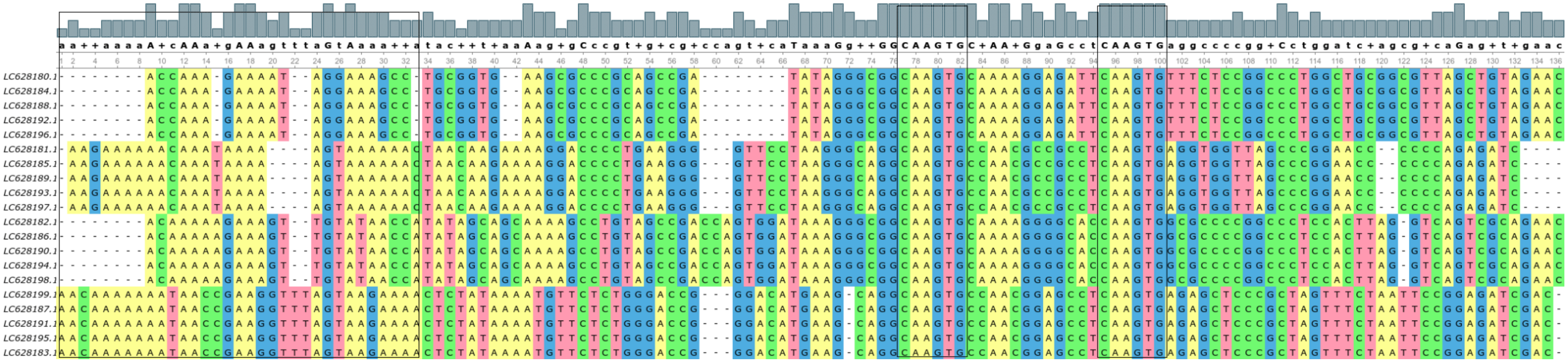

Figure S7. Conserved sequences in the 3′UTRs of Takachi virus genomes found in GenBank according to ClustalW alignment;
